# Comparative analysis of airborne fungal spore distribution in urban and rural environments of Slovakia

**DOI:** 10.1007/s11356-024-35470-5

**Published:** 2024-10-30

**Authors:** Matúš Žilka, Michal Hrabovský, Jozef Dušička, Eva Zahradníková, Dominika Gahurová, Jana Ščevková

**Affiliations:** Department of Botany, Faculty of Natural Sciences, Comenius University, Révová 39, 811 02 Bratislava, Slovakia

**Keywords:** Airborne fungi, Hirst-type sampler, Seasonal spore integral, Cyclone sampler, Amplicon-based metagenomic analysis, Meteorological parameters

## Abstract

Monitoring airborne fungal spores is crucial for public health and plant production since they belong to important aeroallergens and phytopathogens. Due to different land use, their concentration can differ significantly between urban and rural areas. We monitored their spectrum and quantity on two geographically close sites with a different degree of urbanisation: Bratislava City and Kaplna Village in Slovakia, located 38 km apart. We recorded the spectrum of airborne fungal spores over a year and confirmed the microscopic results by amplicon-based metagenomic analysis. The main spore season of the most frequent genera lasted over a week longer in Kaplna, but its intensity was approximately two-fold higher in Bratislava. This can be possibly connected to the microclimatic conditions of the urban area (especially wind speed and heat island effect) and the lesser use of fungicides. *Cladosporium* was the dominant genus on both sites, influencing the intensity most significantly. Through statistical analysis of the influence of meteorological parameters on airborne fungal spore levels, we identified a significant relationship with temperature, while the impact of other parameters varied depending on the spore type and release mechanism. Our results show the differences in airborne fungal spore levels between urban and rural areas and highlight the necessity for more monitoring stations in various environments.

**Supplementary Information:**

The online version contains supplementary material available at 10.1007/s11356-024-35470-5.

## Introduction

Fungi, with more than 150 thousand formally described species (Phukhamsakda et al. 2022) thrive in different environments (Grossart et al. 2019; Debeljak and Baltar 2023). Their successful spread to almost every terrestrial ecosystem has been largely due to airborne spore dispersal (Dijksterhuis 2019). In general, fungal spores constitute about 5% of the PM_10_ mass, though this percentage can rise to as much as 35% in tropical rainforest regions (Elbert et al. 2007; Fröhlich-Nowoisky et al. 2009; Huffman et al. 2012). Most fungal spores are dispersed near the source (Sesartic and Dallafior 2011) but some could be transported over long distances (Sadyś et al. 2014; Grewling et al. 2022).

Despite the undoubtedly positive role of fungi in ecosystems due to their ability to decompose organic matter, several fungi with airborne spores belong to important phytopathogens and aeroallergens, negatively affecting agricultural sustainability and public health. On plants, such as potatoes (*Solanum tuberosum* L.), common grapes (*Vitis vinifera* L.), or various deciduous trees, fungal spores can initiate a variety of diseases such as powdery mildew, rust, and blight, which compromise plant health and productivity (e.g. Dai et al. 2007; Leiminger and Hausladen 2012; Solairaj et al. 2022; Scott et al. 2022). These diseases can lead to substantial agricultural losses by reducing crop yields, increasing the need for chemical fungicides, and diminishing the overall quality of produce. Fungal spores are also common allergens that can trigger respiratory allergic diseases, such as rhinitis, asthma, and alveolitis, in susceptible individuals (Denning et al. 2006; Crameri et al. 2014; D'Amato et al. 2023). More than 80 genera of fungi are associated with allergic sensitisation (Pashley and Wardlaw 2021), with 120 fungal allergen molecules listed in the WHO/IUIS allergen database (http://allergen.org/index.php), predominantly from the two major fungal groups: Ascomycota and Basidiomycota. Ten percent of the global population is sensitive to allergenic fungal spores (Khachatourians 2019).

The species spectrum and concentration of fungal spores in the air depend on several factors, such as meteorological conditions, geographic location, the availability of proper substrates for mycelial growth, and anthropogenic activities (Grinn-Gofroń and Bosiacka 2015; Sztandera-Tymoczek and Szuster-Ciesielska 2023). Among meteorological factors, air temperature, relative humidity, precipitation, and wind speed are the most significant (Grinn-Gofroń et al. 2018). The level of urbanisation influences many of these factors (Kasprzyk and Worek 2006; Oliveira et al. 2010; Liu et al. 2019; Haas et al. 2023), especially substrate availability and anthropogenic activities, but also wind speed and temperature due to the urban heat island effect and associated turbulent air currents (Wang et al. 2019).

Despite extensive research on the temporal distribution of airborne fungal spores in urban environments (Herrero et al. 2006; Klaric and Pepeljnjak 2006; Almaguer et al. 2014; Pyrri and Kapsanaki-Gotsi 2015; Sadyś et al. 2016a, b; Dey et al. 2019; Antón et al. 2019; Núñez et al. 2019; Grinn-Gofroń et al. 2020; Anees-Hill et al. 2022), it is still unknown whether and to what extent their spectrum and concentration differ in geographically close urban and rural environments. Moreover, in most cases, the identification of fungal spores was solely based on their morphological characteristics, which is a less accurate method compared to molecular approaches (Simović et al. 2023). To fill this gap, we aimed to assess the biodiversity and abundance of airborne fungal spores based on their morphology and confirm the results by next-generation sequencing (NGS). The spores were sampled in two areas with different levels of urbanisation: an urban area of Bratislava City and a rural area of Kaplna Village, both located in the Podunajská nížina Lowland, an agriculturally important area of Slovakia. Additionally, the effects of meteorological factors on abundantly represented fungal spore types in the air of both sites were assessed.

## Materials and methods

### Study area

**Fig. 1:**
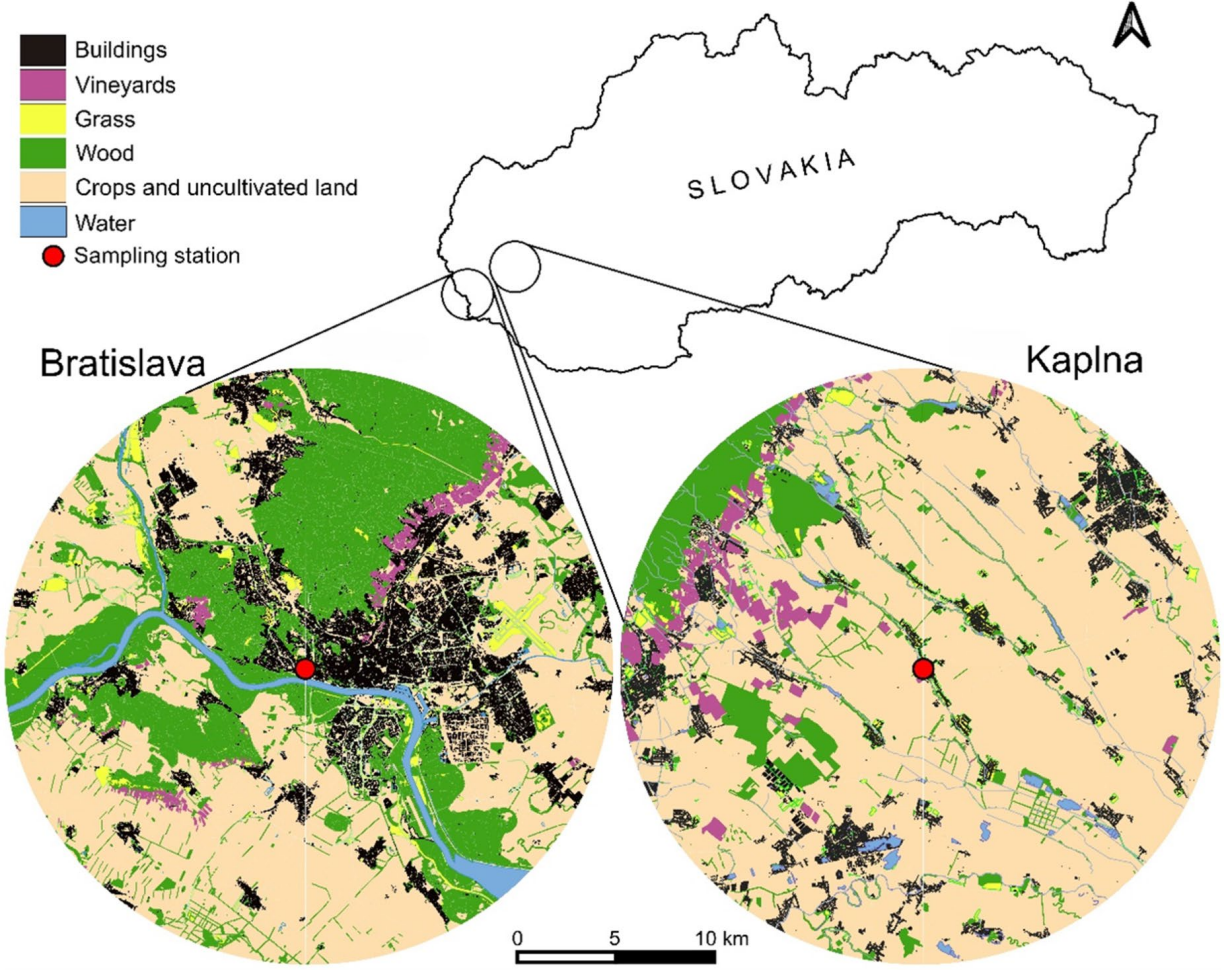
Location of study sites and sampling stations of Bratislava and Kaplna

Airborne bioparticles were sampled from February to November 2022 at two sites differing in urbanisation level and land use. Bratislava (BA), a city in southwestern Slovakia (48° 08′ N, 17° 06′ E, 126–514 m a. s. l.), represented an urban location, and Kaplna (KP), a village 38 km northeast of BA (48° 17′ N, 17° 27′ E, 146 m a. s. l.), represented a rural location (Fig. 1). BA, with 476,922 inhabitants and an area of 367.7 km^2^, exhibits diverse land use. While an extensive part of the city is covered with buildings and anthropogenic structures, urban greenery constitutes approximately 40% of the city area. This greenery primarily consists of Carpathian oak-hornbeam forests, Danube riparian vegetation, and vineyards (Belčáková et al. 2022). The north part of the city extends into the Malé Karpaty Mts. with Devín gate in their northwestern part, affecting air circulation in BA. The southern and eastern parts of the city lie in the Podunajská nížina Lowland, predominantly featuring cultivated arable land. KP is a small rural village, with 944 inhabitants and an area of 5.5 km^2^, situated at the western end of the Podunajská nížina Lowland (~ 150 m a.s.l.). The village area features mainly family houses surrounded by garden vegetation, while the surrounding area is predominantly agricultural, with rapeseed, wheat, and corn as the main crops.

Both study sites are located in Central Europe, where the climate is temperate continental, featuring warm summers and cold winters. In BA, the average annual air temperature is 11.2 °C. July is the warmest month, averaging 21.8 °C, while January is the coldest, with an average temperature of 0.3 °C. The annual total precipitation averages 765 mm, with August being the rainiest month at 87 mm and April the driest at 45 mm (data from 2002 to 2021, collected by the Meteorological Observatory BA–Mlynská dolina located approximately 0.5 km northwest of the monitoring station). The average annual air temperature in KP is 10.6 °C, with July being the warmest month at 21.4 °C and January the coldest at − 0.7 °C. The average annual total precipitation is 579 mm, with August being the rainiest month (68 mm) and April the driest (31 mm) (data from 2002 to 2021, collected at the Meteorological Observatory Jaslovské Bohunice, 26 km southeast of the monitoring station).

In 2022, meteorological conditions varied significantly between the study areas. The average daily air temperature was 11.9 °C in BA and 11.4 °C in KP. BA also experienced substantially higher annual precipitation of 830 mm compared to 455 mm in KP.

### Sample collection and evaluation

Airborne bioparticles were collected at each site using a Hirst-type sampler and a multi-vial cyclone sampler (Burkard Manufacturing Co., Ltd.), which are suitable for microscopic and genetic analyses. The samplers were positioned on building rooftops without obstructions to airflow: 18 m above ground level in BA (N 48.14973, E 17.07375) and 3 m above ground level in KP (N 48.29548, E 17.45022). The lower sampling height in KP was due to the logistical limitations. The operation period of the Hirst-type and cyclone samplers was from 1 February to 31 October 2022. Samples collected by Hirst-type samplers were used for light microscopy to evaluate daily fungal spore diversity and abundance. In this sampler, airborne particles are suctioned through a 2 × 14 mm slit at an airflow rate of 10 L/min and adhere to a strip of adhesive tape moving at 2 mm/h. The tape was changed weekly at the same time, precisely cut into segments representing each sampling day, and mounted on microscopic slides using a mixture of gelatin, glycerin, phenol, and distilled water. The samples were stained with fuchsin to enhance pollen and spore differentiation. Fungal spores were identified using reference atlases (Grant Smith 2000; Lacey and West 2006; Li et al. 2023) and counted under a light microscope (Motic B1-252SP) at × 400 magnification. Each slide was examined along 12 vertical transects (Galán et al. 2021). Daily concentrations were reported as spores per cubic metre of air (spores/m^3^).

The main spore season (MSS) and its characteristics, including season start, end, duration, seasonal spore integral (SSIn), peak value, peak date, and the number of high days (HD), were defined for taxa with annual total spore concentration (annual spore integral (ASIn)) exceeding 2000 spores*day/m3 at both stations (19 taxa). We used the 90% method (Nilsson and Persson 1981) to define the MSS. SSIn was defined as the sum of daily spore concentrations over the MSS. A high day (HD) was defined as a day when the spore concentration exceeded a daily average of 3000 spores/m3 for *Cladosporium* and 100 spores/m3 for other spore taxa. These threshold values are known to trigger clinical fungal spore allergy symptoms (Gravesen 1979; Rapiejko et al. 2007; Sadyś et al. 2016a, b).

Samples collected by multi-vial cyclone samplers were analysed for fungal presence and diversity using amplicon-based metagenomic analysis (hereinafter referred to as metagenomic analysis). The multi-vial sampler operates at an airflow rate of 16.5 L/min, separating particles in a mini cyclone and depositing them into 1.5-mL test tubes, each representing a full day of exposure. The test tubes were stored at − 20 °C until the DNA isolation. Twelve samples (six for each station) were selected for metagenomic analysis. To ensure a high yield of eDNA, we picked the dates with the highest fungal spore diversity and quantity based on the microscopic analysis. These samples were collected on the following dates: BA: 19 June, 16 July, 15 August, 19 August, 14 September, and 18 October and KP: 27 June, 27 July, 12 August, 19 August, 28 September, and 2 October. DNA extraction, Illumina library construction of the amplified internal transcribed spacer region 2 (ITS2) from fungal species present in the aerobiological samples, and a metagenomic study characterising the community using these amplified regions were performed by Microsynth AG (Balgach, Switzerland). ITS2 was amplified with locus-specific primers ITS3 and ITS4 (White et al. 1990) as part of the Nextera barcoded PCR libraries, which were then sequenced on an Illumina MiSeq platform. The paired-end reads that passed Illumina’s chastity filter were de-multiplexed and trimmed of Illumina adapter residuals, and their quality was checked using FastQC software version 0.11.8. The ITS2 primers were trimmed from the sequencing reads using Cutadapt v3.2. The trimmed reads were merged, and fungal ITS2 subregions were extracted with ITSx v1.1.3 and its accompanying database. The obtained Amplicon sequence variants (ASV) sequences also called zero-radius Operational taxonomic units (zOTU) were compared to reference sequences from the UNITE (v7.2) database. Taxa were predicted, and their confidences were calculated using the SINTAX algorithm implemented in USEARCH. The metagenome was visualised using Krona charts, and alpha diversity calculations and rarefaction analysis were performed with the R software packages phyloseq v1.30.0 and vegan v2.5–7.

### Data evaluation

The spore calendars for BA and KP were created using the method described by Spieksma (1991). Mean daily spore concentrations for 10-day periods were categorised into six exponential classes (EC, spore concentrations for *Cladosporium* are in parentheses): (a) 1–10 (1–300), (b) 11–50 (301–1,500), (c) 51–100 (1501–3000), (d) 101–500 (3001–5000), (e) 501–1000 (5001–10,000), and (f) ˃ 1000 (˃ 10,001). The spore calendar only includes the spore types with ASIn reaching 250 + spores/m^3^, listed in chronological order based on the intensity of the MSS.

We categorised the spores based on the period of the year when their average 10-day spore concentrations exceeded the threshold value EC “d”. Seasonal spores are characterised by exceeding the threshold value continuously for over a month and can be categorised as summer, summer-autumn, and autumn spores. When the average 10-day spore concentrations exceed the threshold value as early as spring and these above-threshold levels persist until autumn, we can refer to them as all-year-round spores.

The statistical differences in ASIn between BA and KP spore concentrations were evaluated using a paired samples Wilcoxon test with the wilcox.test() function.

Non-parametric Spearman’s correlation analysis was employed to assess the relationship between the 19 most abundant taxa and selected meteorological variables: daily average mean air temperature (°C), relative humidity (%), wind speed (m/s), sunshine duration (h), and total daily precipitation (mm). The data analyses were conducted using Statistica 12.

## Results

### Diversity of fungal spores in the air of the study areas

Based on microscopic analysis, we recorded 67 fungal spore groups, with 64 present in BA and 59 in KP (Table 1). The taxa confirmed by metagenomic analysis were placed in these groups, together with other potential taxa identified based on their morphology and distribution (Table S1). Except for the Myxomycetes of the Protozoa and *Peronospora* of the Chromista kingdom, all identified spores belong to the Ascomycota or Basidiomycota phylum of the Fungi kingdom, with Ascomycota being the dominant group (Table 1). The most frequently occurring group (> 90% days per year) on both sites were *Alternaria*, *Arthrinium*, *Cladosporium*, *Coprinus* type, *Epicoccum*, and *Leptosphaeria* type. The less frequently occurring groups (< 10% days per year) were *Camarosporium*, *Camptophora*, *Chalastospora*, *Oncopodiella*, *Saccobolus*, *Tetraploa*, and *Tilletia* in BA and *Corynespora*, *Exosporiella* type, *Oncopodiella*, *Panaeolina*, *Sporidesmium*, and *Tilletia* in KP (Table 1)*.*

**Table 1:** Annual Spore Integrals (ASIn), percentage contribution and frequency of all fungal spore types found in the atmosphere of Bratislava and Kaplna in 2022

Spore type	TG	Bratislava			Kaplna		
		ASIn (spore*day/m^3^)	%	Frequency (% of days)	ASIn (spore*day/m^3^)	%	Frequency (% of days)
*Agaricus* type	A	170,831	7.21	68.4	25,243	2.27	69.6
*Agrocybe*	B	9489	0.4	85.4	6843	0.61	89.9
*Alternaria*	A	33,766	1.43	96.9	46,019	4.13	96.4
*Amphisphaeria*	A	1835	0.08	56.5	1365	0.12	52.9
*Arthrinium*	A	2641	0.11	90.1	13,194	1.19	94.6
*Ascobolus*	A	68	< 0.01	17	28	< 0.01	12
*Ascochyta*	A	5342	0.23	34.7	2846	0.26	70.7
*Aspergillus/Penicillium*	A	2141	0.09	37.1	6827	0.61	63.8
*Asterosporium*	A	–	–	–	25	< 0.01	10.1
*Bipolaris*	A	264	0.01	49	84	0.01	12.7
*Botrytis*	A	1764	0.07	50.3	1051	0.09	59.8
*Bovista*	B	716	0.03	34.7	23,855	2.14	63
*Caloplaca*	A	1610	0.07	31	362	0.03	25
*Camarosporum*	A	47	< 0.01	6.8	–	–	–
*Camptophora*	A	4	< 0.01	1	–	–	–
*Cercospora*	A	968	0.04	26.9	595	0.05	33.3
*Cerebella*	A	537	0.02	22.4	452	0.04	27.5
*Cladosporium*	A	1,649,583	69.65	100	726,757	65.25	100
*Coprinus*	B	7367	0.31	85	5317	0.48	80.8
*Coprinus* type	B	172,948	7.3	98	124,548	11.18	96
*Corynespora*	A	–	–	–	6	< 0.01	1.4
*Cucurbitaria*	A	1152	0.05	37.8	–	–	–
*Curvularia*	A	71	< 0.01	18.4	299	0.03	24.3
Diatripaceae	A	3839	0.16	32.7	1920	0.17	21.4
*Drechslera*	A	120	0.01	22.4	213	0.02	44.2
*Epicoccum*	A	14,863	0.63	95.9	11,453	1.03	97.5
*Exosporiella* type	A	309	0.01	35.7	28	< 0.01	3.6
*Exosporium*	A	10,482	0.44	66.3	1523	0.14	71.4
*Fomes*	B	8714	0.37	28.6	–	–	–
*Fusarium*	A	3864	0.16	16.7	3543	0.32	60.9
*Fusicladium*	A	351	0.02	60.5	135	0.01	39.9
*Ganoderma*	B	29,157	1.23	68.7	22,072	1.98	79.3
Helicospores	A	119	0.01	21.8	742	0.07	42.4
*Chaetomium*	A	796	0.03	74.8	1005	0.09	75.4
*Chalastospora*	A	20	< 0.01	2.7	–	–	–
*Leptosphaeria* type	A	108,775	4.59	99.3	22,642	2.03	94.6
*Massaria*	A	47	< 0.01	13.6	223	0.02	43.5
*Melanospora*	A	143	0.01	36.1	341	0.03	57.2
Myxomycetes	P	29,574	1.25	79.9	25,141	2.26	96.4
*Neohendersonia*	A	52	< 0.01	16.3	144	0.01	41.7
*Nigrospora*	A	1326	0.06	92.2	721	0.07	84.4
*Oidium* type	A	53,575	2.26	68	12,263	1.1	81.2
*Oncopodiella*	A	24	< 0.01	8.2	2	< 0.01	1.1
*Panaeolina*	B	175	0.01	21.4	1	< 0.01	0.7
*Panaeolus*	B	611	0.03	48.6	453	0.04	48.9
*Periconia*	A	3041	0.13	94.2	2366	0.21	86.6
*Peronospora*	Ch	722	0.03	67.7	1071	0.1	75.4
*Pithomyces*	A	2889	0.12	73.8	1596	0.14	71.7
*Pleospora*	A	4069	0.17	95.9	4074	0.37	82.6
*Polythrincium*	A	312	0.01	45.6	128	0.01	34.8
*Puccinia*	B	147	0.01	12.9	930	0.08	77.5
*Saccobolus*	A	7	< 0.01	2	–	–	–
*Sordaria*	A	154	0.01	38.8	291	0.03	56.5
*Spegazzinia*	A	73	< 0.01	19	27	< 0.01	10.1
*Splanchonema*	A	176	0.01	34.4	–	–	–
*Sporidesmium*	A	194	0.01	44.6	28	< 0.01	8
*Sporormiella*	A	75	< 0.01	22.4	78	0.01	15.6
*Stemphylium*	A	2726	0.12	62.6	1937	0.17	79.7
Teliospores	B	–	–	–	30	< 0.01	12.3
*Tetraploa*	A	1	< 0.01	0.3	7	< 0.01	25.7
*Tilletia*	B	4	< 0.01	1.4	149	0.01	3.6
*Torula*	A	2639	0.11	85.4	3226	0.29	92.4
Uredinospores	B	2104	0.09	43.5	498	0.05	53.3
*Urocystis*	B	170	0.01	25.5	129	0.01	24.6
*Ustilago*	B	6774	0.29	65.6	1582	0.14	29
*Venturia*	A	304	0.01	21.4	–	–	–
Xylariaceae	A	11,706	0.49	90.5	5436	0.49	77.5
Total		2,368,367	100		1,113,862	100	

*TG* taxonomic group, *A* Ascomycota, *B* Basidiomycota, *P* Protozoa, *Ch* Chromista

**Fig. 2:**
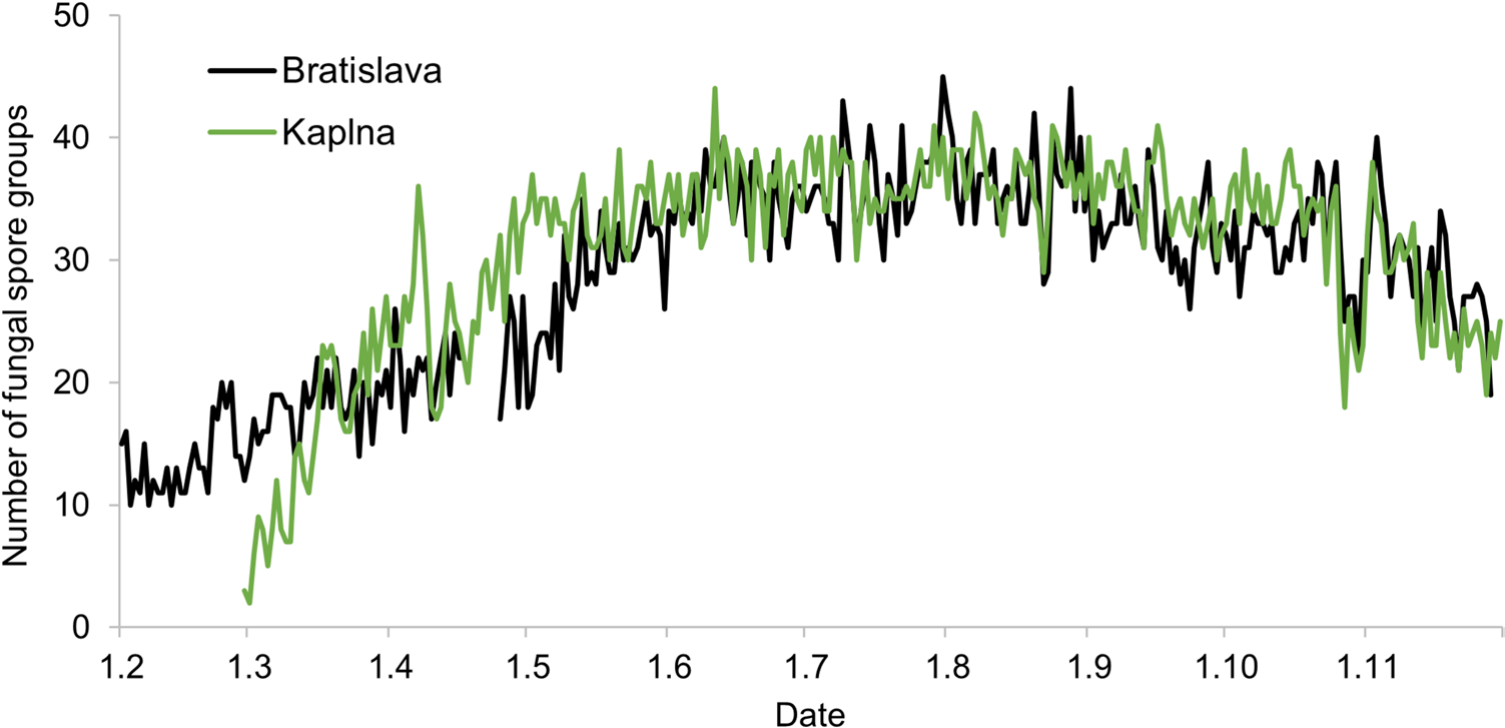
Number of fungal spore groups in the air of Bratislava (BA) and Kaplna (KP) in 2022

The mean daily number of identified spore groups was 28 for BA and 31 for KP. The diversity rose during the spring (March–May), peaked in the summer (June–August), and decreased in autumn (September–November) (Fig. 2). The highest diversity of fungal spores in both areas was recorded in July, with an average number of spore groups of 36 for BA and 37 for KP, and the lowest in February (14 spore types) for BA and in March (15 spore types) for KP.

The results of the metagenomic analysis focused on fungal taxonomic profiling of samples revealed in a total of 96 OTU (operational taxonomic unit) sequences belonging to the phyla Ascomycota and Basidiomycota (Table S2). Ascomycota accounted on average for 84% of fungal DNA in BA and 73% in KP. Of the 10 classes identified, Dothideomycetes and Tremellomycetes were the most abundant. Twenty-three taxa were classified to the order level, with Capnodiales and Pleosporales as the most numerous, and 36 were assigned to the family level and 42 to the genus level. The most abundant genera were *Mycosphaerella*, *Cladosporium*, *Alternaria*, and *Ascochyta.* Of the 40 identified species, *Mycosphaerella tassiana* was present in samples most frequently.

**Fig. 3:**
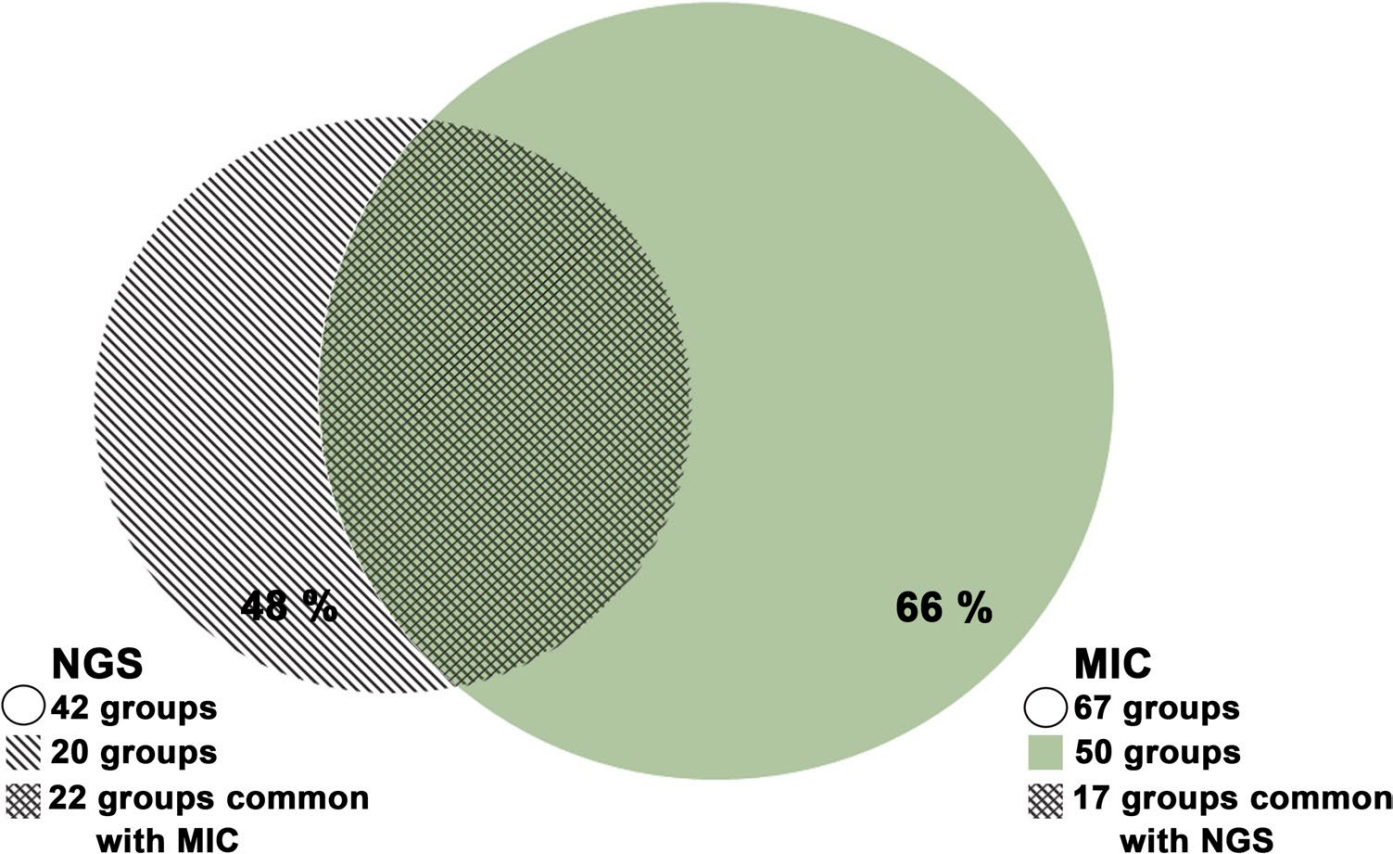
Venn diagram representing the total number of unique and common fungal genera in the airborne samples collected from Bratislava and Kaplna in 2022 based on metagenomic (NGS) and microscopic (MIC) analysis

The metagenomic analysis confirmed the presence of 22 genera which also were detected by microscopic analysis and could be placed in 17 spore groups (Table S1). Forty-eight percent of genera were unique to the metagenomic analysis, and 66% of spore groups were unique to microscopic analysis (Fig. 3).

**Fig. 4:**
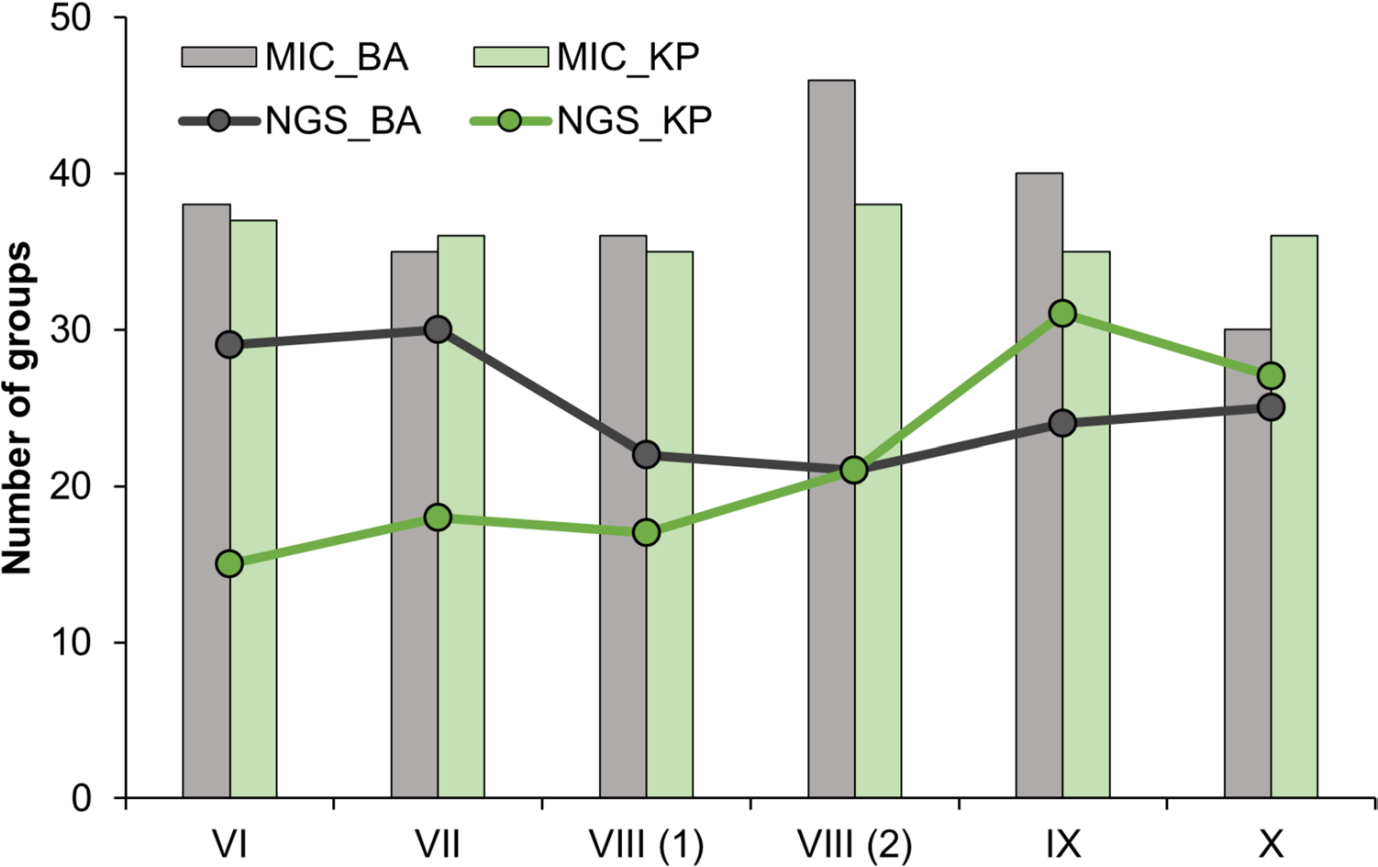
Daily numbers of spore groups recorded by microscopic (MIC) and metagenomic (NGS) analysis during 6 days (VI—19 June, VII—16 July, VIII (1)—15 August, VIII (2)—19 August, IX—14 September, X—18 October) in Bratislava (BA) and 6 days (VI—27 June, VII—27 July, VIII (1)—12 August, VIII (2)—19 August, IX—28 September, X—2 October) in Kaplna (KP) in 2022

The number of genera in each sample from the metagenomic analysis ranged from 21 to 30 for BA and between 17 and 27 for KP. Microscopic analysis of samples from the same dates revealed similar diversity between sites, differing by a maximum of 5 genera (Fig. 4).

### Quantity and abundance of fungal spores in the air of the study areas

**Fig. 5:**
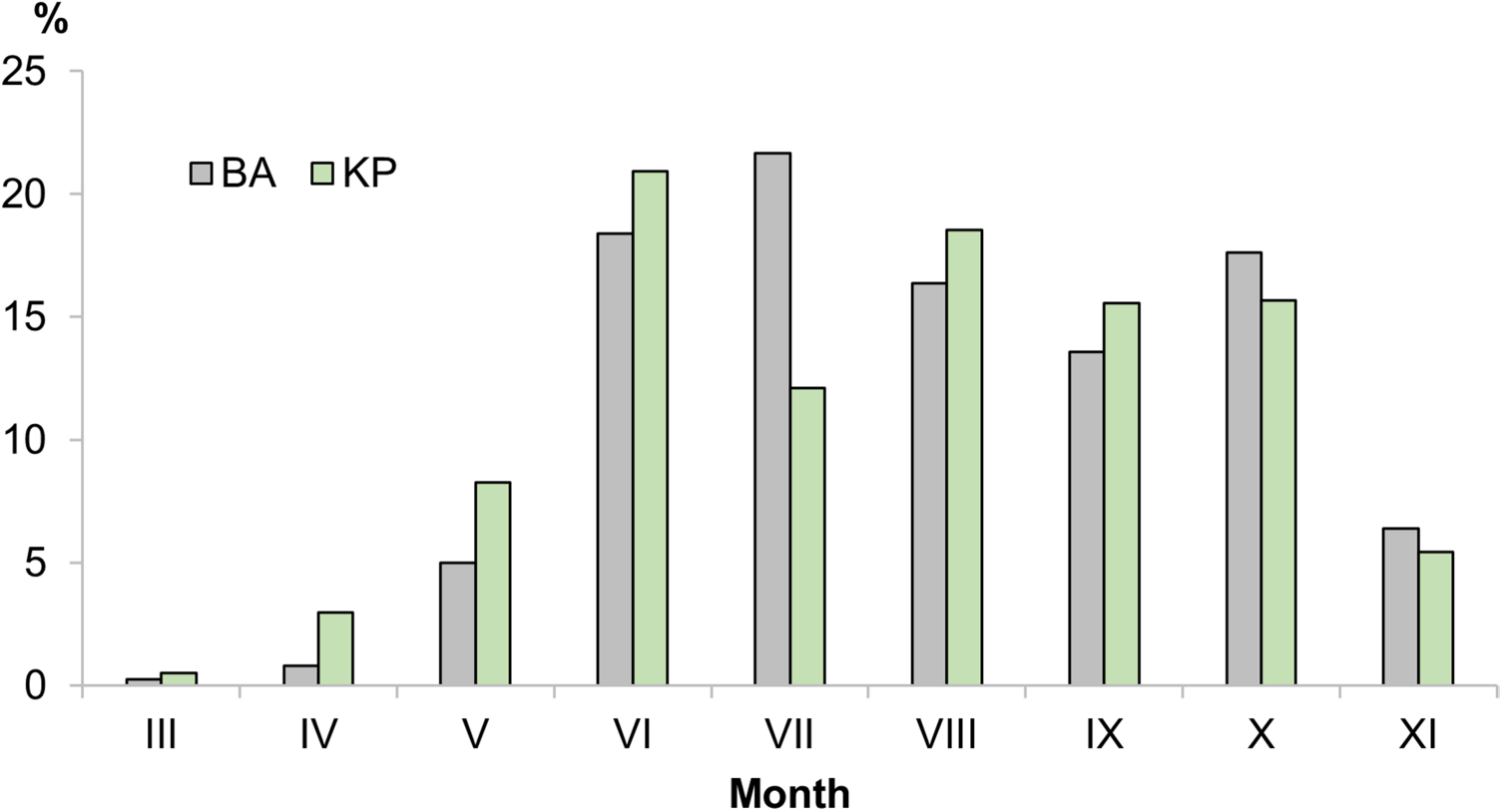
Monthly variation in airborne fungal spore concentration (expressed in percentages) in Bratislava (BA) and Kaplna (KP) in 2022

Based on the results of microscopic analysis, the sum of ASIn for all analysed taxa reached a value of 2,368,367 spore*day/m^3^ in BA and 1,113,864 spore*day/m^3^ in KP (Table 1). Except for winter and early spring months, high concentrations of spores were consistently present in the air, especially in July (21.6% of the total annual spore concentration) for BA and June (20.9%) for KP (Fig. 5). Summer months (June–August) accounted for more than 50% of the ASIn on both sites. Differences in total monthly spore concentration between the two sites were most pronounced in July when over three times more spores were recorded in BA (509,937 spores/m^3^) compared to KP (134,880 spores/m^3^).

The most abundant genera, which accounted for more than 90% of the sum of ASIn, were *Cladosporium*, *Coprinus* type, *Agaricus* type, *Leptosphaeria* type, *Oidium* type, *Alternaria*, Myxomycetes, and *Ganoderma* on both sites. Among the top 10 most abundant taxa were also *Bovista* and *Arthrinium* in KP and *Epicoccum* and Xylariaceae in BA (Table 1). The dominant genus on both sites was *Cladosporium*, contributing 69.6% to the sum of ASIn in BA and 65.2% in KP, followed by *Coprinus* type (BA 7.3%, KP 11.2%). The ratio between genera was similar in study sites, although all the most abundant genera, except for *Alternaria*, had higher concentrations in BA, frequently by two or more-fold (Table 1).

### MSS-related characteristics

The MSS-related characteristics were calculated for the 19 taxa whose ASIn exceeded 2000 spore*day/m^3^ on both sites (Table 2). According to the Wilcoxon test, there was a statistically significant difference in ASIn between BA and KP (*V* = 1644.5, *p* = 0.0016). Regarding timing, MSS for the majority of fungal spore groups started earlier in KP than in BA and ended at approximately the same time in both areas, from mid-August to mid-November. However, the MSS of the most abundant genus *Cladosporium* started 32 days earlier in BA. The first genus to sporulate in both regions was *Periconia* (3 March in BA and 19 March in KP), and the last spore group was the *Agaricus* type (16 September in BA and 8 August in KP). Out of the total evaluated taxa, 11 reached peak value earlier in KP than in BA. In terms of duration, the MSS of most spore groups lasted longer in KP, with the MSS of 11 taxa more than 5 days longer. The duration of MSS ranged from 46 days (*Agaricus* type) in BA to 236 days (*Pleospora*) in KP. The intensity of MSS, expressed by SSIn values, peak value, and number of HD, was higher in BA. The average SSIn value (117,480 spore*day/m3) and peak value (3845 spores/m3) were 133% and 44% higher in BA than in KP, respectively. In BA, 14 taxa achieved a higher SSIn value, and 10 taxa achieved a higher peak value compared to KP. The highest number of HD in BA was recorded for the spore groups *Cladosporium* (151 days), *Coprinus* type (150 days), and *Leptosphaeria* type (144 days), while in KP, it was for *Coprinus* type (122 days), *Alternaria* (120 days), and *Ganoderma* (84 days). The average daily concentration of all 19 spore groups was also higher in BA than in KP except for *Alternaria*, *Aspergillus*/*Penicillium*, *Arthrinium*, *Fusarium*, and *Torula* (Fig. S1). The daily concentration of *Agaricus* type, *Ascochyta*, *Cladosporium*, *Leptosphaeria* type, Myxomycetes, and *Oidium* was most notably higher in BA.

**Table 2:** The MSS characteristics of abundantly represented fungal taxa in the air of Bratislava (BA) and Kaplna (KP) in 2022

Taxa	Season start (DOY)		Season end (DOY)		Season length (days)		SSIn (spore*day/m^3^)		Peak value (spore/m^3^)		Peak day (DOY)		High days^a^ (number)	
	BA	KP	BA	KP	BA	KP	BA	KP	BA	KP	BA	KP	BA	KP
*Agaricus* type	259	219	304	292	46	73	154,457	22,851	14,517	2494	289	281	46	36
*Agrocybe*	163	159	321	318	159	159	8545	6162	410	273	277	306	28	16
*Alternaria*	170	166	299	297	130	131	30,394	41,414	820	1668	239	261	90	120
*Arthrynium*	74	119	295	289	222	170	2386	11,863	48	1885	222	161	0	19
*Ascochyta*	133	117	307	303	175	186	4796	2526	565	115	137	258	14	1
*Aspergillus*/*Penicillium*	126	86	295	262	170	176	1888	6119	146	279	217	97	3	0
*Cladosporium*	96	128	323	303	228	175	1,632,471	653,380	41,841	32,541	187	178	151	77
*Coprinus*	162	131	318	322	157	191	6586	4790	342	336	279	305	17	13
*Coprinus* type	144	130	311	308	168	178	155,746	111,616	5985	5336	279	247	150	122
*Epicoccum*	166	166	307	308	142	142	13,363	10,333	516	456	306	261	46	32
*Fusarium*	159	116	262	312	104	196	3452	3194	357	162	240	68	13	6
*Ganoderma*	169	164	295	300	127	136	26,194	19,862	549	433	224	257	102	84
*Leptosphaeria* type	134	116	307	303	174	187	9810	20,339	3,984	826	183	212	144	73
Myxomycetes	236	130	312	315	77	185	26,221	22,711	1,652	2488	286	290	50	53
*Oidium* type	164	113	226	230	63	117	48,264	11,023	6110	709	184	131	41	30
*Periconia*	62	77	293	307	232	230	2712	2133	74	60	281	142	0	0
*Pleospora*	91	90	326	298	236	208	3643	3697	118	467	329	270	2	8
*Torula*	74	83	289	290	216	207	2377	2901	60	79	170	132	0	0
Xylariaceae	167	160	308	312	142	152	10,533	4871	464	160	259	259	34	7

*SSIn* seasonal spore integral, *DOY* day of the year from 1 January

^a^Spore concentration ˃ 3000 spores/m^3^ for *Cladosporium* and ˃ 100 spores/m^3^ for all taxa except *Cladosporium*

### Spore calendars

**Fig. 6:**
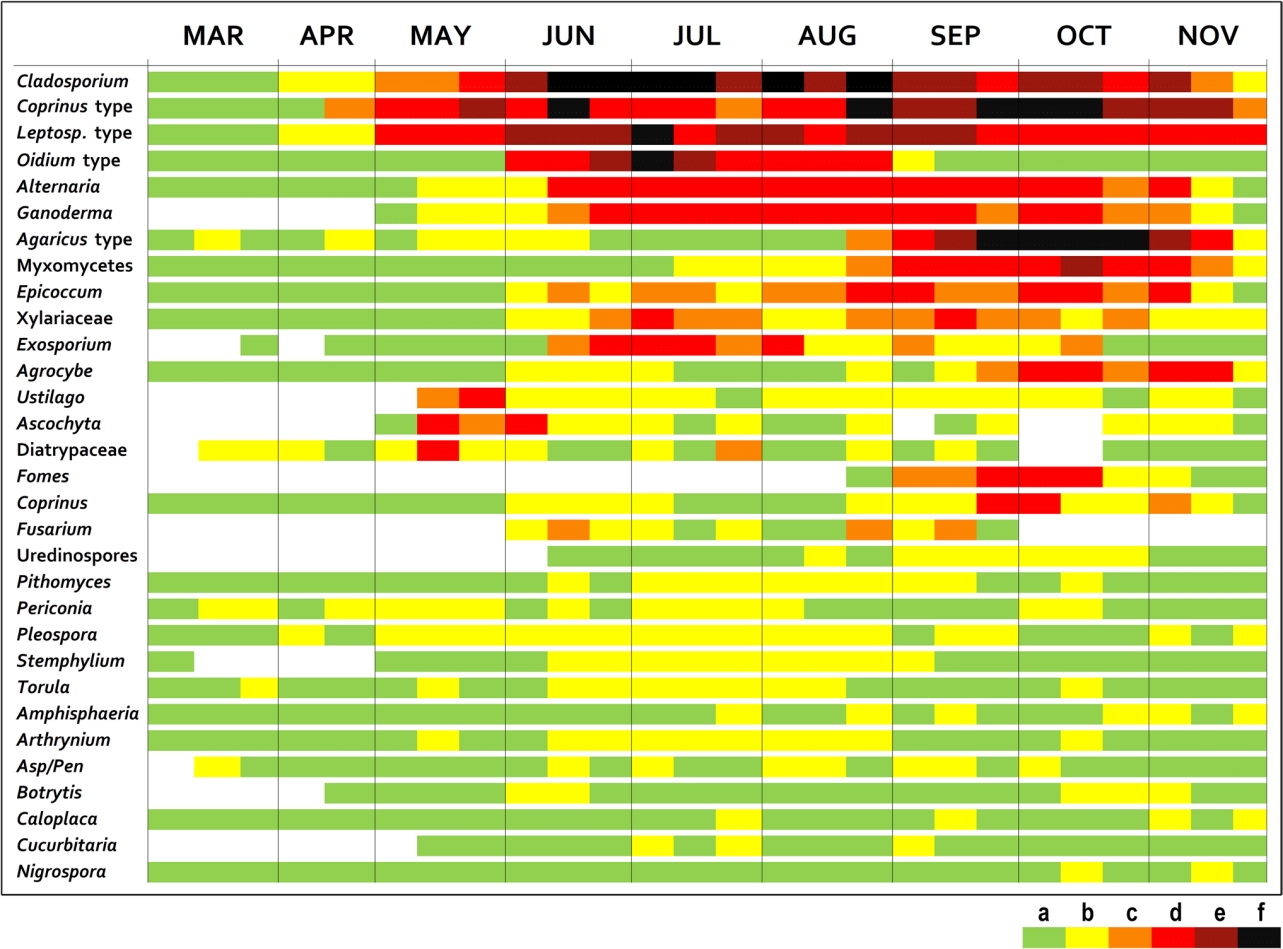
Fungal spore calendar for Bratislava, year 2022. Exponential classes (spores/m^3^): **a** 1–10 (1–300), **b** 11–50 (301–1500), **c** 51–100 (1501–3000), **d** 101–500 (3001–5000), **e** 501–1000 (5001–10,000), **f** ˃ 1000 (˃ 10,001). Spore concentrations for *Cladosporium* are in parentheses. *Asp/Pen*—*Aspergillus*/*Penicillium*; *Leptosph.* type—*Leptosphaeria* type

**Fig. 7:**
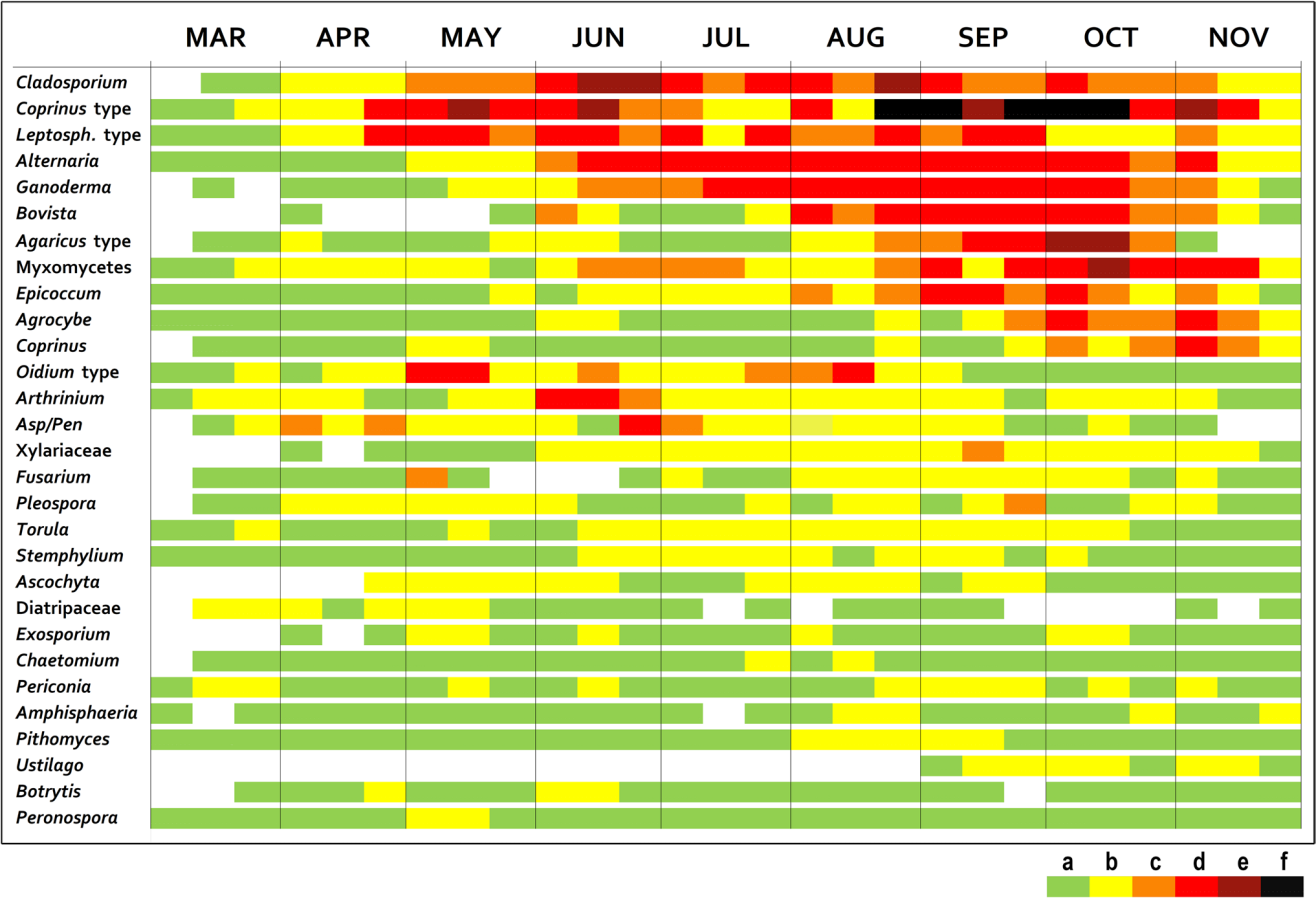
Fungal spore calendar for Kaplna, year 2022. Exponential classes (spores/m^3^): **a** 1–10 (1–300), **b** 11–50 (301–1500), **c** 51–100 (1501–3000), **d** 101–500 (3001–5000), **e** 501–1000 (5001–10,000), **f** ˃ 1000 (˃ 10,001). Spore concentrations for *Cladosporium* are in parentheses. *Asp/Pen*—*Aspergillus*/*Penicillium*; *Leptosph.* type—*Leptosphaeria* type

The fungal spore calendars for BA and KP are shown in Figs. 6 and 7, respectively. For both BA and KP, *Cladosporium*, *Coprinus* type, and *Leptosphaeria* type were identified as all-year-round spores. The *Oidium* type is among the significant summer spores in BA, with concentrations continuously exceeding threshold levels from early June to late August. *Alternaria* and *Ganoderma* are classified as summer-autumn spores, with their concentrations continuously exceeding threshold levels from June except for *Ganoderma* in KP (July) and lasting until the autumn months. Among the significant autumn spores with continuous above-threshold levels between September and November are the *Agaricus* type and Myxomycetes in both locations and *Bovista* in KP. Short-term occurrences of above-threshold levels were also recorded for the spore groups *Epicoccum*, *Agrocybe*, and *Coprinus* in both locations; Xylariaceae, *Exosporium*, *Ustilago*, *Ascochyta*, Diatripaceae, and *Fomes* in BA; and *Aspergillus*/*Penicillium*, *Arthrinium*, and *Oidium* type in KP. In BA, spore concentrations reaching exponential class “f” were recorded for *Coprinus* type, *Leptosphaeria* type, *Agaricus* type, *Oidium* type (above 1000 spores/m3), and for *Cladosporium* (above 10,000 spores/m3). In KP, only the *Coprinus* type reached this value (from the end of August to mid-October). During the sporulation season of *Cladosporium*, we recorded two periods where the concentration exceeded 5000 spores/m3 (exponential class “e”) per day.

### Relationship between airborne spore concentrations and meteorological parameters

For the 19 abundantly represented taxa at both sites, Spearman ‘s correlation analysis identified relative humidity, temperature, and sunshine as the most significant meteorological parameters (Table 3). Relative humidity had both positive and negative effects on fungal spores; however, positive correlations were more frequent and stronger. Strong negative correlations were only observed for *Torula* on both sites and *Ganoderma* and *Arthrinium* in BA. The correlation with temperature was positive for most analysed taxa except for *Agaricus* type, *Agrocybe*, and *Coprinus* in KP. *Alternaria*, *Cladosporium*, *Ganoderma*, and *Torula* had the strongest correlations with temperature on both sites (*p* < 0.001). Correlations with sunshine were similar to those with temperature but weaker. Regarding precipitation, we observed a significant positive relationship with only four spore groups (*Leptosphaeria* type and *Pleospora* at both sites and *Ascochyta* and Diatripaceae in BA). Wind speed was the weakest factor affecting airborne fungal spore concentrations, showing a negative correlation with *Coprinus* type on both sites and with *Ganoderma* and Xylariaceae in BA. A positive influence of the wind speed was only recorded for Diatrypaceae in KP.

**Table 3:** Spearman’s correlation coefficients between airborne fungal spore concentrations and meteorological variables recorded in Bratislava (BA) and Kaplna (KP) in 2022. Significant trends are in bold

Spore group	Temperature (°C)		Sunshine (h)		Relative humidity (%)		Wind speed (m/s)		Precipitation (mm)	
	BA	KP	BA	KP	BA	KP	BA	KP	BA	KP
*Agaricus* type	− 0.056	− 0.224*	− 0.136	− 0.247*	0.294**	0.308**	− 0.141	− 0.133	− 0.104	− 0.077
*Agrocybe*	− 0.191	− 0.242*	− 0.238*	− 0.268**	0.437***	0.486***	− 0.183	− 0.128	− 0.124	− 0.038
*Alternaria*	0.501***	0.433***	0.185	0.095	− 0.008	− 0.072	− 0.093	0.102	− 0.113	− 0.055
*Arthrinium*	0.443***	0.164	0.297**	0.134	− 0.271**	− 0.069	− 0.061	0.117	− 0.097	0.039
*Ascochyta*	− 0.024	0.021	− 0.095	− 0.169	0.124	0.233*	0.041	0.067	0.511***	0.184
*Aspergillus/Penicillium*	0.212*	0.036	0.017	0.022	− 0.088	− 0.134	− 0.077	− 0.058	− 0.045	0.029
*Cladosporium*	0.615***	0.519***	0.291**	0.233*	0.046	− 0.032	− 0.173	0.019	− 0.004	0.078
*Coprinus*	− 0.123	− 0.308**	− 0.234*	− 0.288**	0.405***	0.468***	− 0.121	− 0.161	− 0.093	− 0.085
*Coprinus* type	0.159	0.074	− 0.014	− 0.134	0.342***	0.306**	− 0.249*	− 0.215*	− 0.095	− 0.006
Diatrypaceae	0.107	− 0.153	− 0.268**	− 0.212*	0.282**	0.267**	− 0.08	0.278**	0.456***	0.193
*Epicoccum*	0.291**	0.154	0.019	− 0.117	0.228*	0.215*	− 0.119	0.017	− 0.112	− 0.06
*Ganoderma*	0.591***	0.405***	0.316**	0.12	− 0.447***	− 0.066	− 0.233*	− 0.125	− 0.166	− 0.024
*Leptosphaeria* type	0.365***	0.211*	− 0.055	− 0.183	0.317**	0.276**	− 0.187	0.067	0.342***	0.411***
Myxomycetes	− 0.116	− 0.021	− 0.187	− 0.014	0.283**	0.11	− 0.035	− 0.115	− 0.114	− 0.064
*Oidium* type	0.337***	0.264**	0.259**	0.222*	− 0.089	− 0.207*	− 0.155	− 0.193	− 0.045	− 0.016
*Periconia*	0.314**	0.112	0.308**	0.145	− 0.255*	− 0.093	− 0.045	0.065	− 0.057	− 0.038
*Pleospora*	0.199*	− 0.033	− 0.207*	− 0.297**	0.333***	0.284**	− 0.101	0.043	0.509***	0.467***
*Torula*	0.426***	0.368***	0.308**	0.222*	− 0.304**	− 0.269**	− 0.091	− 0.061	− 0.154	− 0.045
Xylariaceae	0.271**	− 0.014	− 0.033	− 0.153	0.171	0.242*	− 0.244*	0.056	0.042	0.071

^*^p < 0.05, **p < 0.01, ***p < 0.001

## Discussion

### Fungal spore diversity

Hirst-type spore sampling in BA and KP and subsequential assessment by light microscopy revealed 67 different spore groups, with 56 represented on both study sites. Due to the wide range of fungal spores detected, the results can be applied in several sectors such as healthcare, agriculture, or forestry. Such a broad-scale analysis of the fungal spore spectrum is important since most studies are often focused only on individual taxa, rarely including 30 or more of them (Herrero et al. 2006; Pyrri and Kapsanaki-Gotsi 2015; Bednarz and Pawłowska 2016; Grinn-Gofroń et al. 2020). Fungal spore diversity varies throughout the year, reaching its peak in summer when the meteorological conditions are optimal for plant and mycelium growth (Anees-Hill et al. 2022). Ascomycota and Basidiomycota were the dominant spore groups, consistent with other studies focusing on spore morphology in this climate region (Kasprzyk and Worek 2006; Bednarz and Pawłowska 2016).

Applying the metagenomic method, our research revealed 96 OTUs in the Ascomycota and Basidiomycota phylum. The average number of taxa did not vary significantly between sites, similar to the light microscopy results. Fungal diversity in the air is considerably more uniform than in soil or water (Wagner et al. 2022). Significant changes in the diversity of fungal spores in the air are observable at distances greater than 100 km (Abrego et al. 2018), whereas our sites were only 30 km apart. At the same time, the number of taxa generally decreases with increasing urbanisation (Abrego et al. 2020). However, our results showed a slight increase in diversity at urban sites, as revealed by microscopic and metagenomic analyses. This could be attributed to the varying land use and, consequently, the substrate diversity in BA, as well as the placement of the sampler at a higher elevation, allowing it to collect spores from a broader area. In contrast to other studies (Pashley et al. 2012; Yamamoto et al. 2012; Niu et al. 2021; Apangu et al. 2022b), we observed higher fungal diversity when using morphological analysis compared to metagenomic methods. Nevertheless, accurate species classification can still be useful for targeted human health protection and the disease prevention of cultural plants.

Using a metagenomic method, we identified 42 genera, in contrast to the 318 genera and 558 genera identified in atmospheric samples from Tianjin, China (Niu et al. 2021), and New Haven, USA (Yamamoto et al. 2012), respectively. Fröhlich-Nowoisky et al. (2009) demonstrated that 70% of the detected species were found in only one sample, suggesting increased diversity with the number of samples examined. Therefore, we assume that increasing the number of samples could lead to the identification of more genera by metagenomic analysis; however, our goal was only to confirm the taxa identified by morphological analysis. Moreover, the method used for collecting and evaluating samples can significantly influence the outcomes of genetic analyses. Insufficient amounts of readable DNA in samples may contribute to the lower number of identified genera. The prevalence of *Cladosporium* and *Alternaria* was confirmed by both microscopic and metagenomic analysis, in agreement with previous studies (Yamamoto et al. 2012; Núñez et al. 2019; Niu et al. 2021; Simović et al. 2023).

### Fungal spore quantity

Significant variations were observed in the total concentrations of airborne fungal spores, with markedly higher levels detected in urban areas. This is notable, as spore concentrations are typically higher in rural areas due to a greater abundance of plant biomass (Kasprzyk and Worek 2006; Oliveira et al. 2009). The reduced spore concentration in KP may be attributed to fungicide application in neighbouring monocultures and gardens (Oliver and Hewitt 2014). Conversely, such treatments are not employed in the BA area due to the presence of multiple nature reserves and water sources. The harvest of crops can also temporarily increase the spore levels in KP, but this effect is short-lasting due to the harvest being usually done in a single day and does not influence the yearly differences significantly.

The different sampling heights could also affect the quantity and diversity of fungal spores in some way, although not significantly. Other studies (Khattab and Levetin 2008; Damialis et al. 2017; Charalampopoulos et al. 2022) point out the differences in spore concentration between samples collected at ground and rooftop level, with a far more abundant presence of spores on ground level. If this effect were significant, we would expect more spores captured in the KP samples placed at the lower height, but the opposite was true. Therefore we can assume that this effect is not significant in the height of at least 3 m above ground level.

Similarly to other studies (Sadyś et al. 2016a, b; Grinn-Gofroń et al. 2020; Simović et al. 2023), the prevalence of *Cladosporium* was most notable, accounting for 70% and 65% of the total fungal spore concentration in BA and KP, respectively. From the dominant fungal spore groups, only *Alternaria* fit the pattern of higher spore levels in rural environments, with a higher ASIn in KP than in BA. Since *Alternaria* club-shaped spores are the least aerodynamic of this group and originate mostly from local sources (Apangu et al. 2022a), we can assume that the differences are caused by airborne spore transport. In this, BA has a relatively unique position caused by topography increasing the wind speed (Polčák and Šťastný 2011), which can explain the discrepancy with results from other cities. Long-distance transport of airborne bioparticles can bring spores from more distant sources than in KP, where local sources prevail in the bioaerosol composition.

A part of the difference between BA and KP can also be attributed to better conditions for aerosolisation and dispersion of fungal spores in BA. Factors such as increased traffic and movement facilitate the dispersal and resuspension of spores (Muafa et al. 2024), aided by the prevalence of concrete surfaces, which are less adhesive than soil, and differences in microclimatic conditions. In rural areas, the air circulation has a daily periodicity with sedimentation of spores during nighttime due to cooling and increased humidity. In urban environments, the heat island effect (Memon et al. 2008; Wouters et al. 2017) causes the near-surface layer of the troposphere to remain dynamic even during the evening and nighttime hours, preventing spore sedimentation (Jones and Harrison 2004). Our results showing the greatest differences in spore concentrations during summer months hint at this influence of the heat island effect as a possible cause. To mitigate urban heat islands, it is good practice to plant public greenery (Werbin et al. 2020) and retain water in parks, green roofs, and other green spaces within the city (Irfeey et al. 2023). This approach would also help increase the areas to which fungal spores could adhere and reduce their resuspension. However, it is important to select the right plants with regard to their allergenic or invasive potential (Domina et al. 2024). Proper storage and disposal of bio-waste, reduced traffic in the city, and planned denser development would also contribute to lower airborne spore concentrations.

### MSS-related characteristics and spore calendars

Spore calendars, conveying the characteristics of the MSS, are valuable tools for easily tracking the temporal variation in airborne spore concentration throughout the year, making them useful in fields such as medicine, agriculture, forestry, and viticulture. The timing, especially the beginning of the MSS, is most important in agriculture and forestry, while the intensity of the MSS, especially the number of HD, is important in allergology. However, there are only a few examples of spore calendars covering the entire fungal spectrum in the air, and these are primarily from urban environments (Gioulekas et al. 2004; Bednarz and Pawłowska 2016; Antón et al. 2019; Dey et al. 2019; Ščevková and Kováč 2019; Symon et al. 2023).

An earlier start of MSS for most fungal spore groups was observed in KP, with a notable exception of *Cladosporium*. An earlier start of the MSS in a rural environment can be expected due to the closeness of local sources of spores (Ruas et al. 2022). From this point of view, it is important to record the timing of MSS in agricultural areas, since a warning issued by a monitoring station in an urban area, even a close one, could be late for effective treatment of crops.

From an allergological point of view, spore calendars are a useful tool for the effective prevalence and treatment of allergies caused by fungal spores (Katotomichelakis et al. 2016; Weryszko-Chmielewska et al. 2018; Anees-Hill et al. 2022). From the identified spores, six can be found in the WHO/IUIS allergen database (http://allergen.org/index.php): *Alternaria*, *Aspergillus*, *Cladosporium*, *Epicoccum*, *Fusarium*, and *Leptosphaeria* type. Despite the shorter duration of MSS in BA, it had a higher intensity in all of its parameters (SSIn, peak value, and number of HD), which agrees with the higher prevalence of fungal allergies in urban environments (Schröder et al. 2015; Kwong et al. 2023). This can have the same possible causes as the higher quantity of fungal spores in BA (see the “Fungal spore quantity” section).

### Influence of meteorological parameters

Our study identified temperature, sunshine, and relative humidity as the most influential meteorological factors affecting spore concentration. These parameters, along with wind speed and precipitation, have also been highlighted as significant in other studies (Ianovici 2016; Grinn-Gofroń et al. 2018; Anees-Hill et al. 2022). However, the relationship between meteorological conditions and various types of fungal spores is highly complex, with some parameters having opposite effects on different genera.

The influence of temperature on spore concentration is generally positive (Almaguer et al. 2014; Grinn-Gofroń and Bosiacka 2015; Sadyś et al. 2015, 2016a, b; Akgül et al. 2016; Grinn-Gofroń et al. 2018; Olsen et al. 2020), as more plant biomass is produced, serving as a substrate for fungal growth (Rodriguez and Redman 1997), and the fungal mycelium also grows faster (Gange et al. 2007) and produces more spores (up to a certain temperature threshold) (Damialis et al. 2015).

The factors influencing the release of spores differ most noticeably across the different spore types. The dividing of spores into groups of dry-released spores, including *Cladosporium*, *Alternaria*, *Epicoccum*, *Drechslera*, and *Ganoderma* (Troutt and Levetin 2001; Ianovici 2016; Grinn-Gofroń et al. 2018), and wet-released spores, such as *Coprinus*, *Leptosphaeria*, *Fusarium*, *Oidium*, or *Botrytis* (Elbert et al. 2007; Antón et al. 2019), has been established in several studies. In “dry-air” spores, the simultaneous influence of increased temperature, wind speed, and low humidity creates favourable conditions for spore detachment and air dispersal (Ianovici 2016). The increase in temperature and sunshine during the day also accelerates the biomass production of plant hosts, which consequently increases the spore production of their fungal pathogens. In our results, this influence is most clearly seen in *Arthinium*, *Ganoderma*, *Oidium* type, *Periconia*, and *Torula*.

On the contrary, “wet-air” spore release is stimulated by increased humidity, closely linked to precipitation (Grinn-Gofroń and Bosiacka 2015). The increased humidity enables these fungi to grow their fruiting bodies faster and also accelerates sporulation, while rainfall contributes to the easier release of their spores into the air (Talley et al. 2002). These spores are usually colourless and are negatively influenced by UV radiation from sunlight (Klaric and Pepeljnjak 2006). Our results showed a positive correlation with humidity or precipitation and a negative correlation with sunshine, particularly pronounced in *Agrocybe*, *Coprinus*, Diatrypaceae, and *Pleospora*.

## Conclusions

We conducted a study comparing fungal spore spectra and quantities in two nearby areas with varying degrees of urbanisation. Metagenomics allowed us to validate our findings obtained by light microscopy, revealing the degree of correctness of taxon identification and numbers at the two sites. We were also able to determine fungi to the species level, which would not have been possible by light microscopy alone. Furthermore, our knowledge of species diversity was enriched by the addition of new taxa whose presence had not previously been detected at the sites. However, several taxa were not detected by metagenomics, probably due to the small number of collections and the insufficient amount of material in samples. A significantly higher quantity of spores and intensity of the MSS was recorded in the urban area, while the duration of the MSS was longer in the rural area. These findings were predominantly influenced by the genus *Cladosporium*, which constituted a substantial portion of airborne fungal bioparticles in both locations. Meteorological analysis revealed temperature as a key factor positively impacting fungal spore levels, while the effects of other meteorological variables depended on spore release mechanisms. The characteristics of the MSS provide crucial insights for allergology and agricultural practices. However, continued monitoring over multiple years is essential to refine predictions. Our results highlight notable spatial variations in fungal spore levels and MSS characteristics even within geographically proximate areas underscoring the need for additional monitoring stations in economically important regions where airborne fungal spore data is crucial.

## Supplementary Information

Below is the link to the electronic supplementary material.

Supplementary file1 (DOCX 253 KB)

## Data Availability

The raw sequence reads from the amplicon-based metagenomic analysis of 12 samples mentioned in this article, along with 2 outlier samples (omitted from the analysis due to low quality), have been deposited in NCBI’s Sequence Read Archive (SRA) under BioProject accession number PRJNA1163380 (https://www.ncbi.nlm.nih.gov/bioproject/PRJNA1163380).
