# Supplementary material for: Comparative analysis of airborne fungal spore distribution in urban and rural environments of Slovakia
Source: Environ Sci Pollut Res Int. 2024 Oct 30;31(54):63145–60. doi: 10.1007/s11356-024-35470-5 (PMC11599331; doi:10.1007/s11356-024-35470-5)

**Supplementary Materials**

*Comparative analysis of airborne fungal spore distribution in urban and rural environments of Slovakia*

Matúš Žilka, Michal Hrabovský, Jozef Dušička, Eva Zahradníková, Dominika Gahurová, Jana Ščevková

**Table S1** List of genera and species aggregated under fungal spore groups based on morphological data (Li et al. 2023) and amplicon-based metagenomic analysis results (marked by an asterisk)

| Group name | Identified taxa contained in the group (telomorf in brackets) |
| --- | --- |
| *Agaricus* type | *Agaricus* L. spp*.*; *Hypholoma capnoides* (Fr.) P. Kumm*.*; *H. fasciculare** (Huds.) P. Kumm*.*; *Schizopora flavipora** (Berk. & M.A. Curtis ex Cooke) Ryvarden; *Stereum hirsutum** (Willd.) Pers. |
| *Agrocybe* | *A. praecox* (Pers.) Fayod; *Inonotus radiatus* (Sowerby) P. Karst. |
| *Alternaria* | *Alternaria* Nees spp*.*; *A. betae-kenyensis** E.G. Simmons; *A. eichhorniae** Nag Raj & Ponnappa; *A. hordeicola** E.G. Simmons & Kosiak; *A. metachromatica** E.G. Simmons; *A. rosae** E.G. Simmons & C.F. Hill |
| *Amphisphaeria* | *A. multipunctata* (Fuckel) Petr.; *Dothiorella vidmadera** W.M. Pitt, Úrbez-Torr. & Trouillas |
| *Arthrinium* | *A. phaeospermum* (Corda) M.B. Ellis |
| *Ascobolus* | *Ascobolus* Senan. & K.D. Hyde spp*.* |
| *Ascochyta* | *Ascochyta rabiei** (Pass.) Labr.; *A. herbicola** (Wehm.) Qian Chen & L. Cai; *A. medicaginicola var. macrospora** (Boerema, R. Pieters & Hamers) Qian Chen & L. Cai*; Neoascochyta graminicola** (Punith.) Qian Chen & L. Cai; *N. exitialis** (Morini) Qian Chen & L. Cai |
| *Aspergillus* | *Aspergillus melleus** Yukawa*; A. ostianus** Wehmer*; A. flavus* Link*; Penicillium* Link *spp.* |
| *Asterosporium* | *A. asterospermum* (Pers.) S. Hughes |
| *Bipolaris* | *Bipolaris sorokiniana** (Saccardo) Shoemaker |
| *Botrytis* | *Botrytis cinerea* Persoon |
| *Bovista* | *B. pila* Berk. & M.A. Curtis |
| *Caloplaca* | *Caloplaca* Th. Fr. spp*.*; *Rinodina* (Ach.) Gray spp*.*; *Physcia adscendens** H. Olivier; *Phaeophyscia orbicularis** (Neck.) Moberg |
| *Camarosporium* | *Camarosporium abietis* M. Wilson & R.B. Anderson |
| *Camptophora* | *Camptophora hylomeconis** (Crous, de Hoog & H.D. Shin) Réblová & Unter. |
| *Cercospora* | *Cercospora* Fresen. ex Fuckel spp*.* |
| *Cerebella* | *C. andropogonis* Ces. |
| *Cladosporium* | *Cladosporium* sensu lato: *Cladosporium* Link spp*.* (*Mycosphaerella* Johanson); *C. exasperatum** Bensch, Summerell, Crous & U. Braun*; Hyalodendriella* Crous spp*.* |
| *Coprinus* | *C. comatus* (O.F. Müll.) Pers. |
| *Coprinus* type | *Coprinus* sensu lato (- *C. comatus*): *Coprinellus* P. Karst. spp.; *Coprinopsis* P. Karst. spp.; *Coprinus* Pers. spp. |
| *Corynespora* | *Corynespora* Güssow spp. |
| *Cucurbitaria* | *Cucurbitaria* Gray spp. |
| *Curvularia* | *Curvularia intermedia** Boedijn spp*.* |
| Diatrypaceae | *Diatrype disciformis* (Hoffm.) Fr. |
| *Drechslera* | *Drechslera* S. Ito spp.; *Helminthosporium* Link spp.; *Pyrenophora tritici-repentis** (Died.) Drechsler |
| *Epicoccum* | *E. nigrum* Link |
| *Exosporiella* type | *Exosporiella* P. Karst. spp.; *Bactrodesmium betulicola* M.B. Ellis; *Brachysporium nigrum* (Link) S. Hughes |
| *Exosporium* | *E. monanthotaxis* Piroz. |
| *Fomes* | *F. fomentarius** (L.) Fr. |
| *Fusarium* | *Fusarium* Link spp. (*Giberella* Sacc. spp.; *Nectria* (Fr.) Fr. spp.) |
| *Fusicladium* | *Fusicladium* Bonord. sensu lato: *Passalora graminis* (Fuckel) Höhn. |
| *Ganoderma* | *G. adspersum** (Schulzer) Donk; *G. lucidum* (Fr.) P. Karst. |
| Helicospores | *Helicoma* Corda spp.; *Helicomyces* Link spp. |
| *Chaetomium* | *Ch. globosum* Kunze |
| *Chalastospora* | *Chalastospora ellipsoidea** Crous & U. Braun |
| *Leptosphaeria* type | *Leptosphaeria* sensu lato: *Leptosphaeria* Ces. & De Not. spp. (*Diplodina* Westend. spp., *Phoma* Sacc. spp.); *Phaeosphaeria* I. Miyake spp.; *Lophiostoma* Ces. & De Not. spp. |
| *Massaria* | *M. anomia* (Fr.) Petr.; *M. inquinans* (Tode) De Not. |
| *Melanospora* | *Melanospora* Corda spp. |
| Myxomycetes | *Fuligo septica* (L.) F.H. Wigg.; *Physarum nutans* Pers.; *Tubifera ferruginosa* (Batsch) J.F. Gmel. |
| *Neohendersonia* | *N. kickxii* (Westend.) B. Sutton & Pollack; *Triadelphia uniseptate* (Berk. & Broome) P.M. Kirk |
| *Nigrospora* | *Nigrospora oryzae** (Berk. & Broome) Petch |
| *Oidium* type | *Monilia* Bonord. spp.; *Oidium* Link spp. (*Uncinula* Lév. spp.; *Erysiphe* R. Hedw. ex DC. spp.); *Aureobasidium pullulans* (de Bary) G. Arnaud; *Diaporthe neoviticola* Udayanga, Crous & K.D. Hyde |
| *Oncopodiella* | *Oncopodiella* G. Arnaud ex Rifai spp.; *Trinacrium robustum* Tzean & J.L. Chen |
| *Panaeolina* | *P. foenisecii* (Pers.) Maire |
| *Panaeolus* | *P. acuminatus* (P. Kumm.) Quél.; *Harzia verrucosa* (Tognini) Hol.-Jech. |
| *Periconia* | *P. byssoides* Pers.; *P. macrospinosa* Lefebvre & Aar. G. Johnson |
| *Peronospora* | *Peronospora* Corda spp.; *Phytophthora* de Bary spp.; *Plasmopara* J. Schröt. spp. |
| *Pithomyces* | *P. chartarum* (Berk. & M.A. Curtis) M.B. Ellis |
| *Pleospora* | *Pleospora* Rabenh. spp. |
| *Polythrincium* | *Polythrincium* J.C. Schmidt & Kunze spp. |
| *Puccinia* | *Puccinia* Pers. spp.; *P. liliacearum* Duby; *P. coronata* Corda |
| *Saccobolus* | *S. depauperatus* (Berk. & Broome) E.C. Hansen |
| *Sordaria* | *S. fimicola* (Roberge ex Desm.) Ces. & De Not. |
| *Spegazzinia* | *S. tessarthra* (Berk. & M.A. Curtis) Sacc. |
| *Splanchnonema* | *S. foedans* (Fr.) Kuntze |
| *Sporidesmium* | *Sporidesmium* Link spp.; *Ellisembia* Subram. spp. |
| *Sporormiella* | *Preussia* Fuckel spp.; *Sporormiella* Ellis & Everh. spp.; *S. australis* (Speg.) S.I. Ahmed & Cain; *S. minima* (Auersw.) S.I. Ahmed & Cain |
| *Stemphylium* | *Stemphylium* *herbarum** E.G. Simmons |
| Teliospores | various species (mainly *Puccinia* Pers. spp. and *Ustilago* (Pers.) Roussel spp.) |
| *Tetraploa* | *Tetraploa* Berk. & Broome spp. |
| *Tilletia* | *Tilletia* Tul. & C. Tul. spp. |
| *Torula* | *T. herbarum* (Pers.) Gray; *Dendryphion comosum* Wallr. |
| Uredinospores | *Melampsorella* (type) J. Schröt*; Puccinia* spp. Pers. spp. |
| *Urocystis* | *Urocystis* Rabenh. ex Fuckel spp. |
| *Ustilago* | *Ustilago* (Pers.) Roussel spp.; *U. nuda* (C.N. Jensen) Rostr.; *U. maydis* (DC.) Corda |
| *Venturia* | *Venturia* Sacc. spp. |
| Xylariaceae | *Xylaria* Hill ex Schrank spp.; *Daldinia* Ces. & De Not. spp.; *Hypoxylon* Bull. spp. |

Li D-W, Magyar D, & Kendrick B (2023) Color atlas of fungal spores: A laboratory identification guide. American CGIH.

**Table S2** List of identified operational taxonomic units (zOTU)

| zOTU ID | Phyl | Class | Order | Family | Genus | Species |
| --- | --- | --- | --- | --- | --- | --- |
| OTU17 | A | Dothideomycetes | Botryosphaeriales | Botryosphaeriaceae | *Dothiorella* | *Dothiorella vidmadera* |
| OTU21 | A | Eurotiomycetes | Chaetothyriales | Chaetothyriaceae | *Camptophora* | *Camptophora hylomeconis* |
| OTU35 | B | Tremellomycetes | Filobasidiales | Filobasidiaceae | *Filobasidium* | *Filobasidium magnum* |
| OTU34 | A | Sordariomycetes | Microascales | Microascaceae | *Microascus* | *Microascus brevicaulis* |
| OTU30 | A | Dothideomycetes | Myriangiales | NA | NA | NA |
| OTU33 | A | Saccharomycetes | Saccharomycetales | Dipodascaceae | NA | NA |
| OTU39 | A | Dothideomycetes | Pleosporales | Pleosporaceae | *Bipolaris* | *Bipolaris sorokiniana* |
| OTU36 | B | Agaricomycetes | Polyporales | NA | NA | NA |
| OTU38 | A | Dothideomycetes | Pleosporales | Pleosporaceae | NA | NA |
| OTU45 | A | Dothideomycetes | Pleosporales | Didymellaceae | *Ascochyta* | *Ascochyta rabiei* |
| OTU66 | A | Eurotiomycetes | Eurotiales | Aspergillaceae | *Aspergillus* | *Aspergillus melleus* |
| OTU48 | A | Dothideomycetes | NA | NA | *Perusta* | *Perusta inaequalis* |
| OTU55 | A | Eurotiomycetes | Eurotiales | Aspergillaceae | *Aspergillus* | *Aspergillus ostianus* |
| OTU56 | A | Eurotiomycetes | Eurotiales | Aspergillaceae | *Aspergillus* | *Aspergillus flavus* |
| OTU60 | A | Dothideomycetes | Pleosporales | NA | *Chalastospora* | *Chalastospora ellipsoidea* |
| OTU57 | A | Dothideomycetes | Pleosporales | Phaeosphaeriaceae | *Phaeosphaeria* | *Phaeosphaeria triglochinicola* |
| OTU80 | A | Dothideomycetes | Pleosporales | Pleosporaceae | *Curvularia* | *Curvularia intermedia* |
| OTU1 | A | Dothideomycetes | Capnodiales | Mycosphaerellaceae | *Mycosphaerella* | *Mycosphaerella tassiana* |
| OTU2 | A | Dothideomycetes | Pleosporales | Pleosporaceae | *Alternaria* | *Alternaria eichhorniae* |
| OTU3 | A | Dothideomycetes | Capnodiales | Cladosporiaceae | *Cladosporium* | *Cladosporium exasperatum* |
| OTU5 | A | Dothideomycetes | Dothideales | Aureobasidiaceae | *Aureobasidium* | *Aureobasidium pullulans* |
| OTU6 | A | Lecanoromycetes | Caliciales | Physciaceae | *Physcia* | *Physcia adscendens* |
| OTU7 | A | Dothideomycetes | Pleosporales | Didymellaceae | *Ascochyta* | *Ascochyta herbicola* |
| OTU9 | A | Dothideomycetes | Pleosporales | Pleosporaceae | *Alternaria* | *Alternaria hordeicola* |
| OTU15 | B | Tremellomycetes | Tremellales | NA | NA | NA |
| OTU14 | B | Tremellomycetes | Filobasidiales | Filobasidiaceae | *Filobasidium* | *Filobasidium wieringae* |
| OTU12 | A | Dothideomycetes | Pleosporales | Pleosporaceae | *Alternaria* | *Alternaria betae-kenyensis* |
| OTU13 | A | Eurotiomycetes | Chaetothyriales | Herpotrichiellaceae | *Exophiala* | NA |
| OTU16 | A | Dothideomycetes | Pleosporales | Pleosporaceae | *Stemphylium* | *Stemphylium herbarum* |
| OTU22 | A | Saccharomycetes | NA | NA | NA | NA |
| OTU17 | B | Tremellomycetes | Tremellales | Tremellaceae | *Cryptococcus* | NA |
| OTU18 | B | Tremellomycetes | Tremellales | Bulleribasidiaceae | *Vishniacozyma* | *Vishniacozyma victoriae* |
| OTU19 | B | Tremellomycetes | Filobasidiales | Filobasidiaceae | *Filobasidium* | *Filobasidium magnum* |
| OTU20 | B | Microbotryomycetes | Sporidiobolales | Sporidiobolaceae | *Sporobolomyces* | *Sporobolomyces roseus* |
| OTU21 | A | Dothideomycetes | Dothideales | Dothideaceae | *Endoconidioma* | *Endoconidioma populi* |
| OTU23 | A | Dothideomycetes | Pleosporales | Pleosporaceae | *Alternaria* | *Alternaria rosae* |
| OTU25 | B | Tremellomycetes | Tremellales | Bulleribasidiaceae | *Vishniacozyma* | *Vishniacozyma carnescens* |
| OTU33 | A | Xylonomycetes | Xylonomycetales | NA | *Symbiotaphrina* | *Symbiotaphrina kochii* |
| OTU24 | A | NA | NA | NA | NA | NA |
| OTU28 | B | Agaricomycetes | Polyporales | Coriolaceae | *Fomes* | *Fomes fomentarius* |
| OTU26 | B | Tremellomycetes | Tremellales | Bulleraceae | *Bullera* | *Bullera alba* |
| OTU32 | A | NA | NA | NA | NA | NA |
| OTU27 | B | Tremellomycetes | Tremellales | Bulleribasidiaceae | *Hannaella* | *Hannaella luteola* |
| OTU37 | B | Agaricomycetes | Agaricales | NA | NA | NA |
| OTU31 | A | NA | NA | NA | NA | NA |
| OTU36 | A | Dothideomycetes | Pleosporales | Pleosporaceae | *Alternaria* | *Alternaria metachromatica* |
| OTU34 | A | Dothideomycetes | Capnodiales | Teratosphaeriaceae | NA | NA |
| OTU35 | A | Saccharomycetes | Saccharomycetales | NA | NA | NA |
| OTU41 | B | Tremellomycetes | Filobasidiales | Filobasidiaceae | *Filobasidium* | *Filobasidium oeirense* |
| OTU39 | A | Leotiomycetes | Helotiales | Sclerotiniaceae | *Botrytis* | *Botrytis cinerea* |
| OTU53 | A | Saccharomycetes | Saccharomycetales | Saccharomycetaceae | *Saccharomyces* | NA |
| OTU38 | A | Dothideomycetes | Pleosporales | Phaeosphaeriaceae | NA | NA |
| OTU40 | A | Dothideomycetes | NA | NA | NA | NA |
| OTU42 | A | Sordariomycetes | Trichosphaeriales | Trichosphaeriaceae | *Nigrospora* | *Nigrospora oryzae* |
| OTU43 | A | Leotiomycetes | NA | Pseudeurotiaceae | NA | NA |
| OTU48 | A | Dothideomycetes | Pleosporales | Didymellaceae | *Neoascochyta* | *Neoascochyta graminicola* |
| OTU46 | A | Dothideomycetes | Pleosporales | NA | NA | NA |
| OTU50 | A | NA | NA | NA | NA | NA |
| OTU45 | B | Tremellomycetes | Tremellales | Bulleribasidiaceae | *Dioszegia* | *Dioszegia hungarica* |
| OTU47 | A | Dothideomycetes | Capnodiales | Mycosphaerellaceae | *Ramularia* | *Ramularia collo-cygni* |
| OTU52 | A | Eurotiomycetes | Eurotiales | Aspergillaceae | *Aspergillus* | *Aspergillus flavus* |
| OTU54 | B | Tremellomycetes | Tremellales | Tremellaceae | *Cryptococcus* | *Cryptococcus laurentii* |
| OTU49 | A | Dothideomycetes | Dothideales | NA | NA | NA |
| OTU55 | A | NA | NA | NA | NA | NA |
| OTU60 | A | NA | NA | NA | NA | NA |
| OTU62 | B | Agaricomycetes | Agaricales | NA | NA | NA |
| OTU63 | A | NA | NA | NA | NA | NA |
| OTU57 | A | Eurotiomycetes | Eurotiales | Aspergillaceae | NA | NA |
| OTU59 | A | Dothideomycetes | Pleosporales | Pleosporaceae | *Pyrenophora* | *Pyrenophora tritici-repentis* |
| OTU66 | A | Xylonomycetes | Xylonomycetales | NA | *Symbiotaphrina* | *Symbiotaphrina buchneri* |
| OTU67 | B | Tremellomycetes | Filobasidiales | Filobasidiaceae | *Filobasidium* | *Filobasidium stepposum* |
| OTU69 | B | Agaricomycetes | Corticiales | Corticiaceae | *Schizopora* | *Schizopora flavipora* |
| OTU76 | B | Agaricomycetes | Agaricales | Strophariaceae | *Hypholoma* | *Hypholoma fasciculare* |
| OTU73 | B | Cystobasidiomycetes | NA | NA | *Buckleyzyma* | *Buckleyzyma aurantiaca* |
| OTU85 | B | Agaricomycetes | Corticiales | Corticiaceae | *Hyphodontia* | *Hyphodontia radula* |
| OTU72 | B | Cystobasidiomycetes | NA | NA | *Symmetrospora* | *Symmetrospora coprosmae* |
| OTU92 | B | Agaricomycetes | Russulales | Peniophoraceae | *Peniophora* | NA |
| OTU78 | B | Agaricomycetes | Polyporales | NA | NA | NA |
| OTU71 | B | Tremellomycetes | Tremellales | Phaeotremellaceae | *Gelidatrema* | NA |
| OTU70 | A | Dothideomycetes | Pleosporales | Montagnulaceae | NA | NA |
| OTU94 | B | Agaricomycetes | Russulales | Stereaceae | *Stereum* | *Stereum hirsutum* |
| OTU74 | A | Eurotiomycetes | Chaetothyriales | Herpotrichiellaceae | *Rhinocladiella* | NA |
| OTU77 | A | Dothideomycetes | Pleosporales | Didymellaceae | *Ascochyta* | *Ascochyta medicaginicola var. macrospora* |
| OTU84 | B | Agaricomycetes | Trechisporales | Hydnodontaceae | *Sistotremastrum* | *Sistotremastrum guttuliferum* |
| OTU87 | B | Agaricomycetes | Polyporales | Coriolaceae | *Trametes* | *Trametes trogii* |
| OTU75 | A | Leotiomycetes | Helotiales | Sclerotiniaceae | *Monilinia* | NA |
| OTU86 | A | Dothideomycetes | Pleosporales | Phaeosphaeriaceae | *Pha*eosphaeria | NA |
| OTU81 | B | Agaricomycetes | Polyporales | Coriolaceae | *Trametes* | *Trametes hirsuta* |
| OTU80 | A | Dothideomycetes | Pleosporales | Didymellaceae | *Neoascochyta* | *Neoascochyta exitialis* |
| OTU90 | A | Dothideomycetes | NA | NA | NA | NA |
| OTU89 | A | Lecanoromycetes | Caliciales | Physciaceae | *Phaeophyscia* | *Phaeophyscia orbicularis* |
| OTU91 | A | Dothideomycetes | Pleosporales | Leptosphaeriaceae | NA | NA |
| OTU88 | A | Dothideomycetes | NA | NA | NA | NA |
| OTU82 | A | Dothideomycetes | Pleosporales | Phaeosphaeriaceae | *Paraphoma* | NA |
| OTU83 | A | NA | NA | NA | NA | NA |
| OTU96 | B | Agaricomycetes | Polyporales | Ganodermataceae | *Ganoderma* | *Ganoderma adspersum* |
| OTU93 | B | Agaricomycetes | Agaricales | Psathyrellaceae | *Coprinellus* | *Coprinellus xanthothrix* |

*Phyl* – Phylum; *A* - Ascomycota, *B* – Basidiomycota; *NA* - unknown

**Fig. S1** Variation in average daily concentrations of most abundant fungal spore groups in Bratislava (BA) and Kaplna (KP) in 2022.

*Asp/Pen* – *Aspergillus*/*Penicillium*


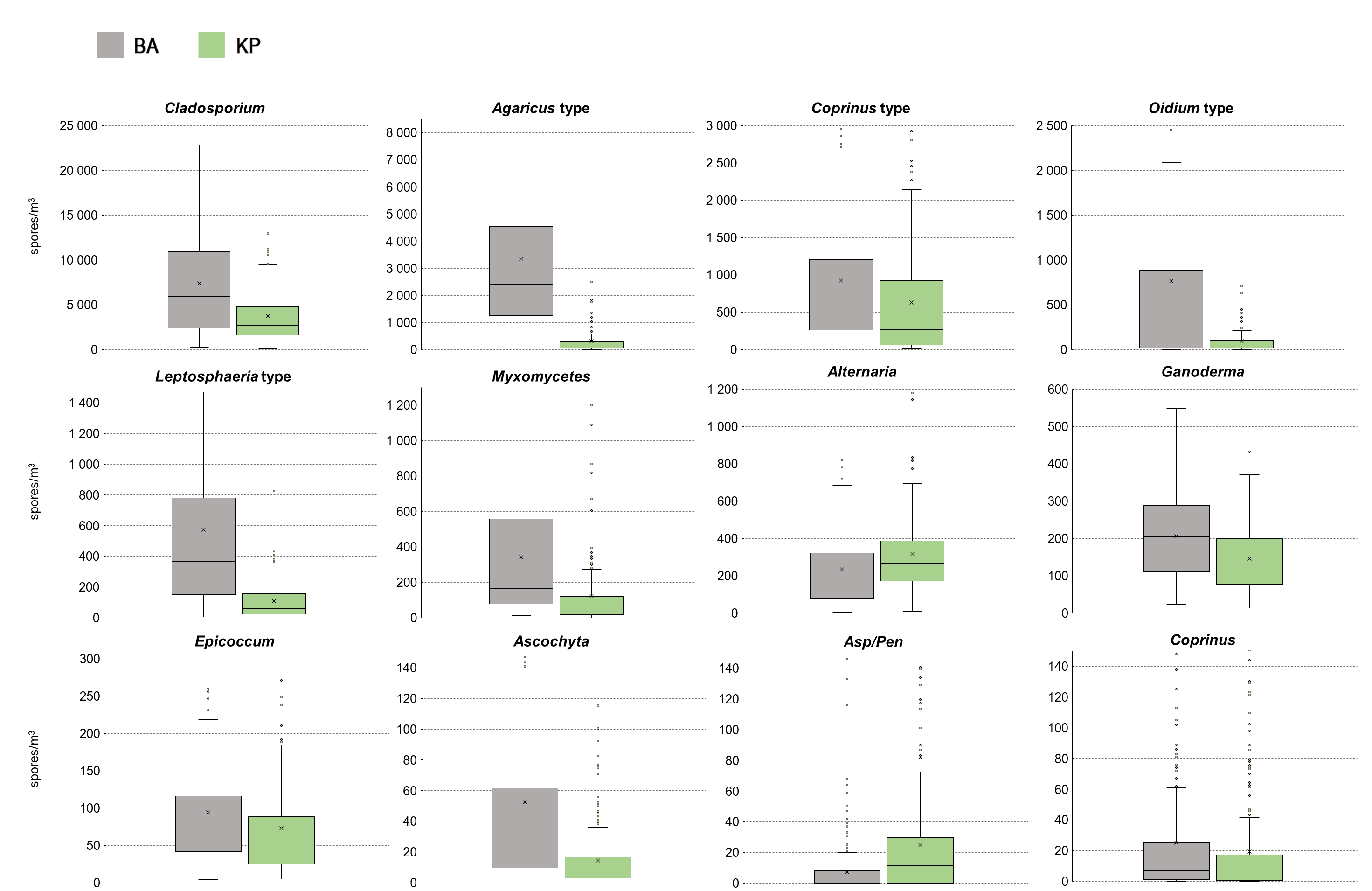


**Fig. S1** continued


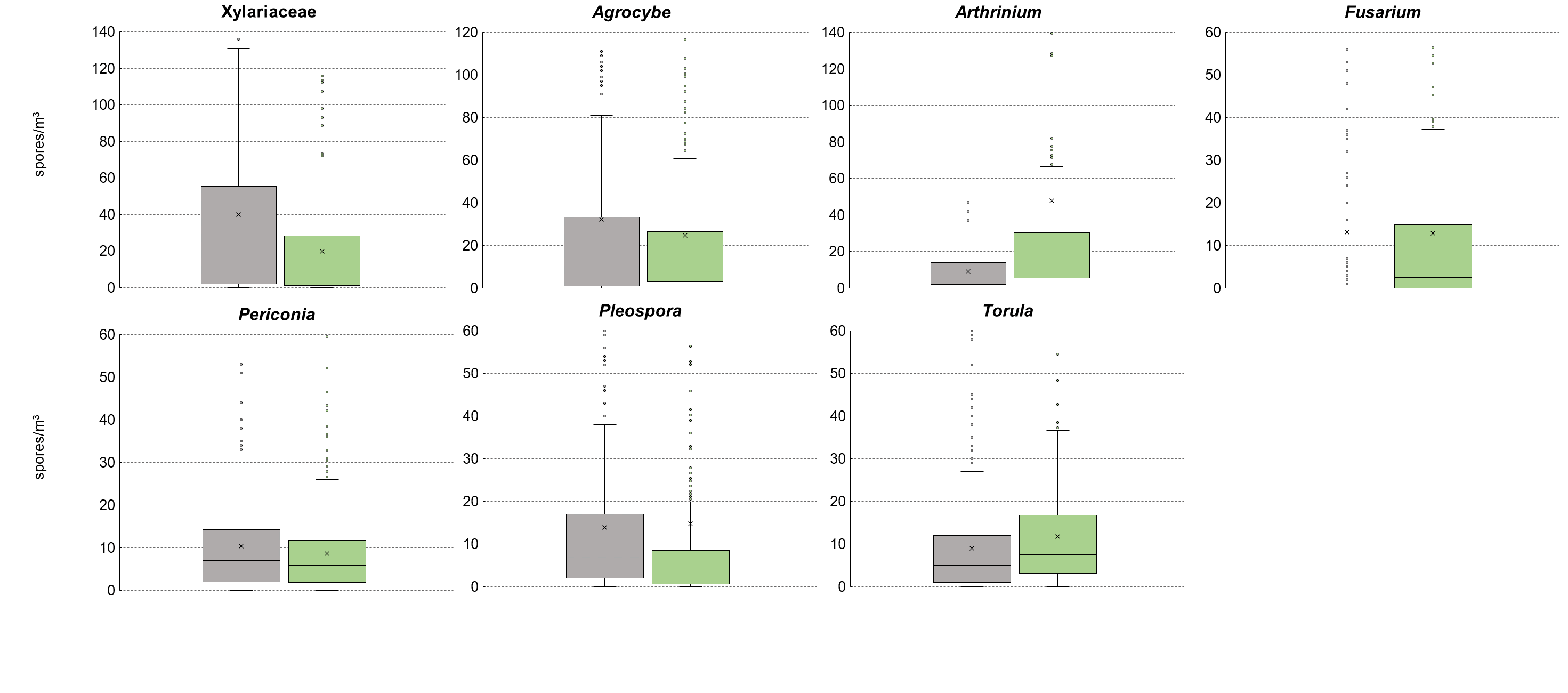

Supplement: Supplementary file 1 — Supplementary file1 (DOCX 253 KB) [file 11356_2024_35470_MOESM1_ESM.docx]
